# Analysis of the impact of COVID-19 variants and vaccination on the time-varying reproduction number: statistical methods

**DOI:** 10.3389/fpubh.2024.1353441

**Published:** 2024-07-03

**Authors:** Geunsoo Jang, Jihyeon Kim, Yeonsu Lee, Changdae Son, Kyeong Tae Ko, Hyojung Lee

**Affiliations:** ^1^Nonlinear Dynamics and Mathematical Application Center, Kyungpook National University, Daegu, Republic of Korea; ^2^Department of Statistics, Kyungpook National University, Daegu, Republic of Korea

**Keywords:** COVID-19, time-varying reproduction number, serial interval, variant, public health intervention, vaccination

## Abstract

**Introduction:**

The COVID-19 pandemic has profoundly impacted global health systems, requiring the monitoring of infection waves and strategies to control transmission. Estimating the time-varying reproduction number is crucial for understanding the epidemic and guiding interventions.

**Methods:**

Probability distributions of serial interval are estimated for Pre-Delta and Delta periods. We conducted a comparative analysis of time-varying reproduction numbers, taking into account population immunity and variant differences. We incorporated the regional heterogeneity and age distribution of the population, as well as the evolving variants and vaccination rates over time. COVID-19 transmission dynamics were analyzed with variants and vaccination.

**Results:**

The reproduction number is computed with and without considering variant-based immunity. In addition, values of reproduction number significantly differed by variants, emphasizing immunity’s importance. Enhanced vaccination efforts and stringent control measures were effective in reducing the transmission of the Delta variant. Conversely, Pre-Delta variant appeared less influenced by immunity levels, due to lower vaccination rates. Furthermore, during the Pre-Delta period, there was a significant difference between the region-specific and the non-region-specific reproduction numbers, with particularly distinct pattern differences observed in Gangwon, Gyeongbuk, and Jeju in Korea.

**Discussion:**

This research elucidates the dynamics of COVID-19 transmission concerning the dominance of the Delta variant, the efficacy of vaccinations, and the influence of immunity levels. It highlights the necessity for targeted interventions and extensive vaccination coverage. This study makes a significant contribution to the understanding of disease transmission mechanisms and informs public health strategies.

## 1 Introduction

The severe acute respiratory syndrome coronavirus 2 (SARS-CoV-2) pandemic represents the most significant global health crisis in recent memory, inflicting an enormous burden on healthcare systems. Since the COVID-19 patient was first reported in December 2019, decisions to tighten or relax restrictions have become a crucial aspect of policymaking. Instead of lockdowns, the Korean government implemented social distancing measures, recommending remote learning for schools, and telecommuting for work (1, 2).

As COVID-19 has spread globally, nations have adopted a range of strategies of non-pharmaceutical (NPIs) and pharmaceutical interventions such as vaccination (3–5). It is crucial to evaluate how these political approaches have influenced the spread of the disease and to forecast the potential impacts of alternative strategies. Numerous studies forecasted the number of COVID-19 cases using the mathematical modeling or stochastic approaches (6, 7). Moreover, several studies incorporated factors like sex, age, and race in predicting COVID-19 cases (8, 9). The characteristics of the two reproduction numbers were simulated using the Susceptible-Exposed-Infectious-Recovered (SEIR) model for countries with similar profiles. Rozhnova et al. (10) utilized an age-structured model for SARS-CoV-2 to analyze hospital admissions and seroprevalence data from spring 2020. Implementing measures focusing on reducing contact outside school was proved to be more effective in reducing time-varying reproduction number ( $R_{t}$ ).

The value of $R_{t}$ is defined as the expected number of secondary cases arising from a primary case infected at time t (11, 12), summarizes the potential transmissibility of a disease, and indicates controllability of the epidemic. $R_{t}$ is an important parameter in public health because it determines the extent of an epidemic. It is a proven, powerful tool for monitoring and tracking epidemics and guiding public health restriction adjustments. This study posits that $R_{t}$ provides an effective way to understand epidemic dynamics during its evolution, as demonstrated in (13), thus aiding the formation of national policies and public health interventions.

Typically, $R_{t}$ changes during an epidemic because of various factors such as the depletion of susceptible individuals, alterations in contact behavior, seasonal patterns of pathogens, and control interventions (3–5). Depending on the country and timing, some studies suggest a significant correlation between climate conditions and the spread (14, 15), while others report minimal or no impact (16, 17). In addition, Alpha, Delta, and Omicron variants have emerged as globally dominant strains of the virus (18, 19). Although non-pharmaceuticals and vaccinations have been implemented, the impact of virus variants is important for understanding the rapid increase in outbreaks. Vaccination was found to be a key tool against serious diseases and deaths, which reduced the burden on medical systems as hospitalization rates among the older adult decreased sharply (20, 21).

In the present study, we considered immune individuals who have experienced the infection from COVID-19 or received a vaccination. The number of individuals with immunity can change over time. Herd immunity may appear temporarily at the peak of the number of cases in the early stages of an epidemic, which can help suppress the epidemic. Due to a significant number of infections and primary and booster vaccination drives in Malaysia, herd immunity has been achieved within the population. Consequently, the value of $R_{t}$ in Malaysia is considerably lower than that in other countries (22). However, this condition does not mean that herd immunity will continue indefinitely. Determining the level of immunity required for group-level inhibition is crucial. Social measures such as social distancing may help contain future waves of the pandemic, but the temporary stability will eventually weaken (23).

To estimate $R_{t}$ , different approaches have been developed and are broadly categorized into two groups: those based on compartmental models (3, 24) and those that directly infer the number of secondary infections per infected individual using a time series of infection incidence (25–27). For the latter category, Cori et al. (26) proposed the EpiEstim method in 2013 using renewal equations, which has now been adopted by numerous studies (12, 27–29).

Serial interval (SI), which refers to the duration between the onset of symptoms in an infected individual and that in a person they infect, is a crucial measure for estimating epidemiological parameters, such as reproduction number, generation time, and attack rate. These parameters are essential for predicting disease trends and assessing healthcare requirements. SI is fundamental for calculating the basic reproduction number ( $R_{0}$ ), which signifies the number of secondary infections resulting from a single infector throughout the entire infectious period (30). These measures are used to forecast disease trajectories and healthcare requirements. Previous studies estimated that the serial intervals of COVID-19 ranged from 3.96 to 5.2 days (30–32).

Previously, $R_{t}$ was estimated using data on the number of reported cases (26). Existing studies estimated $R_{t}$ by assuming SI to follow specific set of values (33, 34). However, in this study, we aim to analyze $R_{t}$ by considering virus variants and vaccinations using SI estimated from data collected from the Republic of Korea.

## 2 Methods

We computed the probability distributions of SI using epidemiological data from Korea. We calculated $R_{t}$ of the COVID-19 variants using the EpiEstim method (26) to understand the impact of vaccination and the effectiveness of control interventions. Additionally, we developed a new time-varying reproductive number without considering immunity ( $R_{v}$ ). Finally, we compared the two types of reproduction numbers; $R_{t}$ as the baseline, including immunity, and $R_{v}$ , without considering immunity. It expands while considering variants ( x ) or regional characteristics ( g ) for each $R_{v}$ and $R_{t}$ .

### 2.1 Epidemiological data

We analyzed epidemiological data on 30,413,435 reported cases of COVID-19 in the Republic of Korea from February 26, 2021 to March 6, 2023, provided by the Korea Disease Control and Prevention Agency (KDCA) (18). The proportions of the Delta and Omicron variants among all COVID-19 reported cases were obtained from the covariance data (35). The time intervals used for our analysis were categorized into three periods, based on the globally dominant variants, which are Delta and Omicron, observed (18, 19), summarized in Supplementary Table S1. In the Republic of Korea, the emergence of COVID-19 cases with the Delta variant began in April 2021, and by 11 July, 2021, it accounted for more than 50% of the total cases (36, 37). Subsequently, from January 11, 2022, the Omicron variant was the predominant strain, representing more than 50% of the cases, resulting in a rapid increase in the number of COVID-19 cases (38). Therefore, we designated the three periods as “Pre-Delta” (February 26, 2021–July 10, 2021), “Delta” (July 11, 2021–January 10, 2022), and “Omicron” (January 11, 2022–March 6, 2023), and the time intervals were labeled as T_Pre-Delta_, T_Delta_, and T_Omicron_, respectively.

We divided the total population into 17 age groups with interval of 5 years, ranging from 0–4 years to 80 years and older. Weekly vaccination data were extracted for the first, second, and third dose vaccinations administered to different age groups, as provided by the KDCA (35). Details on the Korean population size, segregated by age group and region for the year 2021, were obtained from Statistics Korea (39).

### 2.2 Estimation of probability distribution of serial interval

For estimating the probability distributions of the SIs, we first calculated the number of transmission pairs in the data based on infector onset dates. To account for the data reported daily, the discretized probability density function $f\left(t,\theta \right)$ was defined at time t for the parameter of the distribution θ . For example, in gamma distribution, the parameter θ represents a vector of mean (*μ*) and standard deviation (SD) (*σ*) of the probability distribution, such that $\theta =\left(\mu ,\sigma \right)$ . Then, the likelihood function for an SI is defined as


(1) $$L\left(\theta ;d\right)=\overset{m}{\underset{i=1}{\prod }}f\left(d\left(i\right);\theta \right)\text{,}$$

where m is the total number of pairs and d indicates the serial intervals for time period (i.e., $d=\left\{d\left(1\right),\ldots ,d\left(m\right)\right\}$ ). We used 70,414 infector–infectee pairs to estimate the SI distribution, while four commonly used distributions for epidemiological periods: gamma, log-normal, normal, and Weibull (40, 41) were employed to estimate the time period. The performance of each statistical model was compared by calculating the Akaike information criterion (AIC).


$$AIC=-2\ln\left(L\right)+2K$$

where *K* is the number of parameters used. Among the four commonly used statistical models, the best-fitted distribution was selected based on the minimum AIC values. $h\left(\tau \right)$ indicates the probability distributions of the SIs estimated from February 18, 2020 to March 6, 2023 during the total period. We defined the best-fitted distribution as $h_{Pre-\mathrm{Delta}}$ and $h_{\mathrm{Delta}}$ for the Pre-Delta period and for the Delta period, respectively.

To consider non-positive values in the SI data, the analysis involved two approaches: fitting the distributions to positive values only (truncated), and fitting the distributions to shifted data with 11-day delays added to each observation (shifted) (42). Thus, we assumed that pre-symptomatic transmissions could be accounted by adding delays to each observation. Hence, a more accurate representation of the underlying distribution was captured and meaningful insights from the data were derived.

### 2.3 Time-varying reproduction number by variants and vaccination

We assessed $R_{t}$ to quantify the time-dependent variations in the average number of secondary cases generated per case during the course of the outbreak due to intrinsic (decline in susceptible individuals) and extrinsic factors, such as behavioral changes and implementation of public health measures (43–45) In Korea, the reported cases vary throughout the week, with notably lower counts observed on Saturday and Sunday. We applied a moving window using a 21-day window to address this variability. By using the smoothed data on COVID-19 cases, we estimated the evolution of $R_{t}$ for COVID-19 in the Republic of Korea.

Several studies estimated the most recent $R_{t}$ by simulating the progression of incident cases and applying the discretized probability distribution of the generated interval using renewal equations (13, 26, 43, 46). In a model study conducted across 131 countries, the impact of implementing and easing eight different NPIs on $R_{t}$ was examined (47). The reopening of schools; lifting of bans on public events, gatherings of 10 or more people, and stay-at-home orders; and easing of internal movement restrictions were found to increase $R_{t}$ . However, the effects of NPI implementation and easing were not immediate. Using maximum likelihood estimation (MLE) and sequential Bayesian methods, $R_{0}$ and $R_{t}$ were estimated (48).

$R\left(t\right)$ was defined as the total number of incident cases $I\left(t\right)$ arising at time *t*, divided by the discretized probability function $h\left(t\right)$ , which was defined at time *t* with the lowest value of AIC for truncated distributions, as shown in Table 1.


(2) $$R\left(t\right)=\frac{I\left(t\right)}{\sum_{\tau =1}^{t}I\left(t-\tau \right)h\left(\tau \right)}$$

**Table 1:** Estimation of probability distribution of serial interval by variants.

Period distribution		Total (*n* = 70,414) Jan 9, 2020–Jan 10, 2022			Pre-Delta (*n* = 29,945) Jan 9, 2020–Jul 10, 2021			Delta (*n* = 40,469) Jul 11, 2021–Jan 10, 2022		
		Mean	SD	AIC	Mean	SD	AIC	Mean	SD	AIC
Truncated	Gamma	3.86	3.48	258,570.53	4.29	3.94	108,318.66	3.57	3.15	149,652.12
	Weibull	3.85	3.41	258,449.04	4.29	3.84	108,247.39	3.56	3.08	149,571.72
	Normal	3.87	3.34	290,269.11	4.30	3.70	120,746.96	3.58	3.03	167,844.64
	Lognormal	4.12	5.02	262,484.48	4.63	5.94	110,149.37	3.79	4.41	151,935.65
Shifted (+11 days)	Gamma	14.61	4.05	393,957.91	14.79	4.59	167,328.05	14.48	3.64	225,008.07
	Weibull	14.53	4.26	397,562.67	14.74	4.65	167,779.40	14.39	3.94	228,278.10
	Normal	14.61	3.93	393,047.72	14.79	4.41	166,698.10	14.48	3.56	224,705.94
	Lognormal	14.67	4.39	400,335.77	14.88	5.06	170,311.35	14.52	3.90	228,298.24

*n* indicates the number of observed serial intervals.

To compute 95% credible intervals (95% CrIs) of $R\left(t\right)$ , the bootstrapping method was applied to generate 100 samples from the Gamma distributions (26).

#### 2.3.1 Time-varying reproduction number by variants

The time intervals were categorized as T_Pre-Delta_, T_Delta_, and T_Omicron_. A reproduction number method was suggested considering Alpha, Beta, Gamma, and Delta multiple variants (49). We computed the number of Pre-Delta, Delta, and Omicron by multiplying the daily COVID-19 cases with proportional data. The proportion of *x* variant at time *t* was defined as $\phi_{x}\left(t\right)$ . $I_{x}\left(t\right)$ indicated the number of COVID-19 cases caused by *x* variant at time *t*, expressed as $I_{x}\left(t\right)=\phi_{x}\left(t\right)\;I\left(t\right)$ , where $x\in X;X=\left\{Pre-\mathrm{Delta},\mathrm{Delta},\mathrm{Omicron}\right\}$ . Due to the lack of data for infector–infectee pairs during Omicron, the probability distribution of the SI for both Delta and Omicron was assumed as $h_{\mathrm{Delta}}=h_{\mathrm{Omicron}}$ , such that


(3) $$R_{x}\left(t\right)=\frac{I_{x}\left(t\right)}{\sum_{\tau =1}^{t}I_{x}\left(t-\tau \right)h_{x}\left(\tau \right)}$$

#### 2.3.2 Time-varying reproduction number by immunity

For our analysis, we considered the evolving nature of the disease based on the number of immune individuals, including those who covered from COVID-19 or received a vaccination. As of June 2023, over 94% of individuals aged 12 years and older were fully vaccinated with the required dose, while more than 60% of the total population was infected. Therefore, for this study, we considered the remaining population who were yet to develop immunity and were susceptible. Several studies suggested estimation of time-varying reproductive number by immunity (50), such that the effect of the *k*-th vaccination against for the dominant *x* variant was represented by $\sigma_{k}^{x}$ , where $x\in X;X=\left\{Pre-\mathrm{Delta},\mathrm{Delta},\mathrm{Omicron}\right\}$ .

The patients were divided into 17 age groups at 5-year intervals, such that $n_{a}=17$ indicated the number of age groups, $N_{a}$ represented the population size of age group *a*, $P_{k,a}\left(t\right)$ defined the population size of the *k*-th vaccination in age group *a* at time *t*, and $V_{k,a}\left(t\right)$ represented the *k*-th vaccination rate in age group *a* at time *t*. Thus, $V_{k,a}\left(t\right)=P_{k,a}\left(t\right)/N_{a}$ . $V_{k}\left(t\right)=\overset{n_{a}}{\underset{a=1}{\sum }}V_{k,a}\left(t\right)$ presented the *k*-th vaccination rate at time *t*. For each variant *x*, the proportion of individuals with immunity at time *t* ( $\rho_{T_{x}}\left(t\right)$ ), during period $T_{x}$ was defined as


$$\rho_{T_{x}}\left(t\right)=\sigma_{1}^{x}V_{1}\left(t\right)+\sigma_{2}^{x}V_{2}\left(t\right)+\sigma_{3}^{x}V_{3}\left(t\right)\;\mathrm{if}\;t\;\in T_{x}\text{.}$$

The proportion of individuals with immunity at time *t* was expressed by $\rho \left(t\right)=U_{x}\rho_{Tx}\left(t\right)$ . Therefore, $R_{v}\left(t\right)$ without immunity was defined as


(4) $$R_{v}\left(t\right)=\frac{I\left(t\right)}{\left(1-\rho \left(t\right)\right)\sum_{\tau =1}^{t}I\left(t-\tau \right)h\left(\tau \right)}$$

Accounting for the dominant variant x without immunity in Eq. (4), $R_{v,x}\left(t\right)$ was defined as


(5) $$R_{v,x}\left(t\right)=\frac{I_{x}\left(t\right)}{\left(1-\rho T_{x}\left(t\right)\right)\sum_{\tau =1}^{t}I_{x}\left(t-\tau \right)h_{x}\left(\tau \right)}$$

#### 2.3.3 Time-varying reproduction number by regions

We grouped the seven geographical regions of Korea as Seoul Metropolitan Area, Gangwon, Chungcheong, Honam, Gyeongbuk, Gyeongnam, and Jeju, such that ( $g=\left\{1,2,\cdots ,7\right\}$ ) shown in Supplementary Figure S1. $N_{a}$ represented the population size of age group *a*, while $N_{a,g}$ defined the population size of age group *a* in region g . $V_{k,g}\left(t\right)$ represented the *k*-th vaccination rate in region g at time *t*, and was defined as


$$V_{k,g}\left(t\right)=\overset{n_{a}}{\underset{a=1}{\sum }}\frac{V_{k,a}\left(t\right)N_{a,g}}{N_{a}}\text{.}$$

For each variant *x*, the proportion of individuals with immunity ( $\rho_{T_{x},g}\left(t\right)$ ) in region g , at time *t*, during period $T_{x}$ was defined as


$$\rho_{T_{x},g}\left(t\right)=\sigma_{1}^{x}V_{1,g}\left(t\right)+\sigma_{2}^{x}V_{2,g}\left(t\right)+\sigma_{3}^{x}V_{3,g}\left(t\right)\;\mathrm{if}\;t\;\in T_{x}\text{,}$$

where the proportion of susceptible individuals in region g at time *t* was expressed as $\rho_{g}\left(t\right)={\;}_{x}\rho_{Tx,g}\left(t\right).\;I_{g}\left(t\right)$ . $I_{g}\left(t\right)$ indicated the number of COVID-19 cases in region g at time *t*, and $I_{x,g}\left(t\right)=\phi_{x}\left(t\right)I_{g}\left(t\right)$ indicated the number of COVID-19 cases in region g , by variant *x*, at time *t*. Therefore, the time-varying reproduction number without considering the immunity in region g by variant *x* was defined as


(6) $$R_{x,g}\left(t\right)=\frac{I_{x,g}\left(t\right)}{\sum_{\tau =1}^{t}I_{x,g}\left(t-\tau \right)h_{x}\left(\tau \right)}$$

Accounting for immunity by the dominant variant x without immunity in Eq. (6), $R_{v,x,g}\left(t\right)$ was defined as


(7) $$R_{v,x,g}\left(t\right)=\frac{I_{x,g}\left(t\right)}{\left(1-\rho_{T_{x},g}\left(t\right)\right)\sum_{\tau =1}^{t}I_{x,g}\left(t-\tau \right)h_{x}\left(\tau \right)}$$

All Equations (2)–(7) are summarized in Table 2. $R_{t}$ refers to the time-varying reproduction numbers with immunity such as $R\left(t\right),R_{x}\left(t\right),R_{x,g}\left(t\right)$ . $R_{v}$ refers to the time-varying reproduction numbers without immunity such as $R_{v}\left(t\right),R_{v,x}\left(t\right),R_{v,x,g}\left(t\right)$ . To compare $R_{t}$ and $R_{v}$ , we employ various statistical measures including maximum, mean, median, minimum, SD, proportion of R>1 , coefficient of variation (CV), which is defined as the ratio of the standard deviation ( σ ) to the mean ( μ ) (i.e., $CV=\frac{\sigma }{\mu }$ ). The proportion of R>1 indicates the number of time points that satisfy when the reproduction number is greater than 1.

**Table 2:** Summary of time-varying reproduction numbers.

	Formula	Description	Eq.
$R_{t}$	$R\left(t\right)=\frac{I\left(t\right)}{\sum_{\tau =1}^{t}I\left(t-\tau \right)h\left(\tau \right)}$	**Time-varying reproduction number with immunity** $R\left(t\right)$ represents the average number of secondary cases at time t .	(2)
	$R_{x}\left(t\right)=\frac{I_{x}\left(t\right)}{\sum_{\tau =1}^{t}I_{x}\left(t-\tau \right)h_{x}\left(\tau \right)}$	**(2) by variants** $R_{x}\left(t\right)$ represents the average number of secondary cases caused by variant x at time t .	(3)
	$R_{x,g}\left(t\right)=\frac{I_{x,g}\left(t\right)}{\sum_{\tau =1}^{t}I_{x,g}\left(t-\tau \right)h_{x}\left(\tau \right)}$	**(3) by regions** $R_{x,g}\left(t\right)$ represents the average number of secondary cases caused by variant x in region g at time t .	(6)
$R_{v}$	$R_{v}\left(t\right)=\frac{I\left(t\right)}{\left(1-\rho \left(t\right)\right)\sum_{\tau =1}^{t}I\left(t-\tau \right)h\left(\tau \right)}$	**Time-varying reproduction number without immunity** $R_{v}\left(t\right)$ represents the average number of secondary cases without immunity at time t .	(4)
	$R_{v,x}\left(t\right)=\frac{I_{x}\left(t\right)}{{\left(1-\rho T_{x}\left(t\right)\right)\sum }_{\tau =1}^{t}I_{x}\left(t-\tau \right)h_{x}\left(\tau \right)}$	**(4) by variants** $R_{v,x}\left(t\right)$ represents the average number of secondary cases caused by variant x without immunity at time t .	(5)
	$R_{v,x,g}\left(t\right)=\frac{I_{x,g}\left(t\right)}{\left(1-\rho T_{x,g}\left(t\right)\right)\sum_{\tau =1}^{t}I_{x,g}\left(t-\tau \right)h_{x}\left(\tau \right)}$	**(5) by regions** $R_{v,x,g}\left(t\right)$ represents the average number of secondary cases caused by variant x in region g without immunity at time t	(7)

Eq. refers to the equation.

$R_{t}$ is the time-varying reproduction number with the immunity as baseline.

$R_{v}$ is the time-varying reproduction number without considering the immunity.

$I\left(t\right)$ is the number of cases at time *t*.

$I_{x}\left(t\right)$ is the number of cases at time *t* by variant x .

$I_{x,g}\left(t\right)$ is the number of cases at time t by variant x and region g .

$1-\rho \left(t\right)$ is proportion of susceptible individuals.

$1-\rho T_{x}\left(t\right)$ is proportion of susceptible individuals of variant x .

$1-\rho T_{x,g}\left(t\right)$ is proportion of susceptible individuals of variant x and region g .

$h\left(\tau \right)$ is probability distribution of SI.

$h_{x}\left(\tau \right)$ is probability distribution of SI of variant x .

## 3 Results

### 3.1 Transmission dynamics of COVID-19 epidemic with variants and vaccination

**Figure 1:**
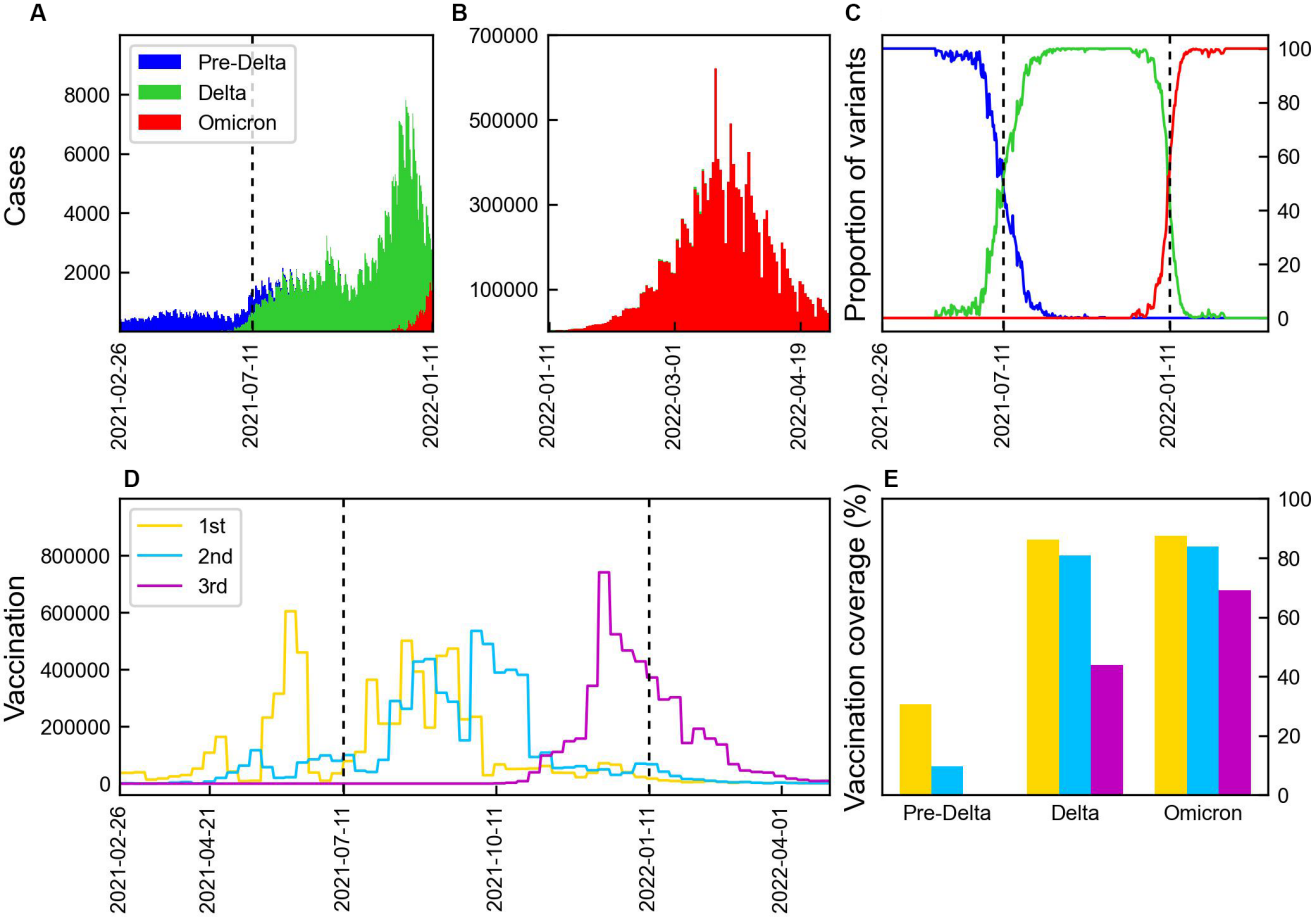
Number of COVID-19 cases by variants and vaccination. The vertical lines indicate the start points of the time periods, T_Delta_ and T_Omicron_. **(A,B)** Number of COVID-19 cases by Pre-Delta, Delta, and Omicron variants over time from February 2021 to April 2022 in Korea. **(C)** Proportion of COVID-19 variation. **(D)** Weekly doses of 1st, 2nd, and 3rd vaccination. **(E)** Vaccination coverage during three periods: 1st (yellow), 2nd (blue), and 3rd (magenta) vaccination.

Data on the number of COVID-19 cases, proportion of variants, and vaccination coverage are presented in Figure 1. Figures 1A,B present the number of confirmed cases caused by Delta and Omicron variants. The number of cases increased with the emergence of new mutations, with the Delta variant causing the highest number of cases, reaching over 8,000. During Omicron, the number of cases peaked at approximately 600,000 before decreasing. The proportion of the variants are shown in Figure 1C, where the dashed lines indicate the start points of the time periods, T_Delta_ and T_Omicron_ during which the proportion of each variant exceeded 50%. The vaccination coverage is shown in Figure 1D. The first and second vaccine doses were administered during Pre-Delta, with the majority receiving the second dose during Delta. The third doses of booster shots were administered 2 months prior to the emergence of the Omicron variant. The vaccination coverage for each period is shown in Figure 1E. During Pre-Delta, the first dose accounted for about 40% of the total population, whereas the second dose accounted for only 10%. During Delta, the first and second doses were administered to 80% of the population. The third dose was administered to 40% of the population during Delta, while 70% were covered during Omicron.

### 3.2 Estimation of probability distribution of serial interval

**Figure 2:**
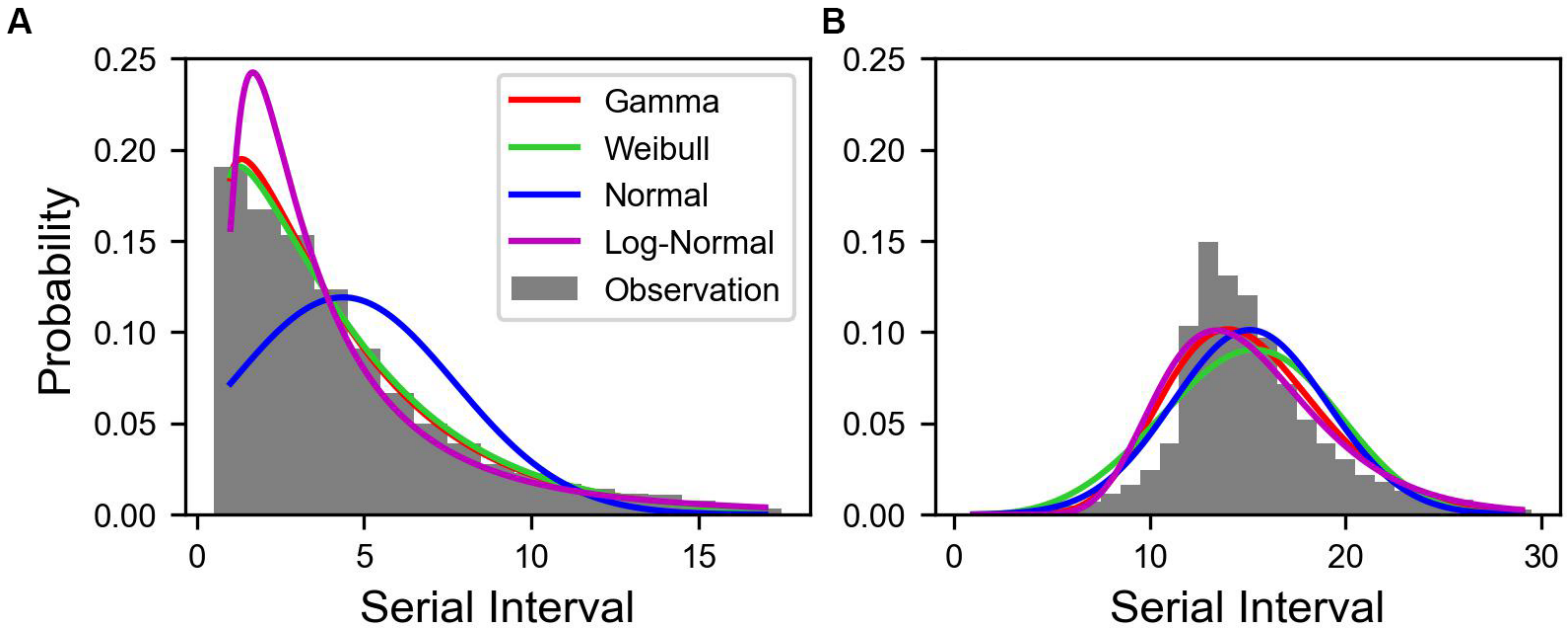
Estimated serial interval distribution of COVID-19 in Korea. Bars indicate the observed data of serial interval from January 9, 2020 to January 29, 2022. The colored lines indicate the estimated serial interval. **(A)** Truncated and **(B)** shifted distributions over 11 days.

Out of 30,413,435 COVID-19 cases, we reconstructed 70,414 transmission pairs from the known onset dates for the infectors and infected population. The SIs ranged from −11 to 17 days, and were estimated using truncated and shifted distributions for the entire period, as shown in Figure 2. Based on the AIC values, the truncated Weibull distribution provided the best fit for all three periods. The estimated mean SI for the total period was 3.85 days, with an SD of 3.41 days, as shown in Figure 2A. To compute $R_{t}$ , we employed the estimated SI using the truncated Weibull distribution for each period (Pre-Delta, Delta), as presented in Table 1. For Pre-Delta and Delta, the estimated mean SIs were 4.29 and 3.56 days, with SDs of 3.84 and 3.08 days, respectively. The estimated SIs for Pre-Delta and Delta from the truncated and shifted distributions are shown in Supplementary Figure S2. The different values of $R_{t}$ obtained from truncated and shifted distributions using the Gamma, Weibull, Lognormal methods are presented in Supplementary Figure S3.

**Figure 3:**
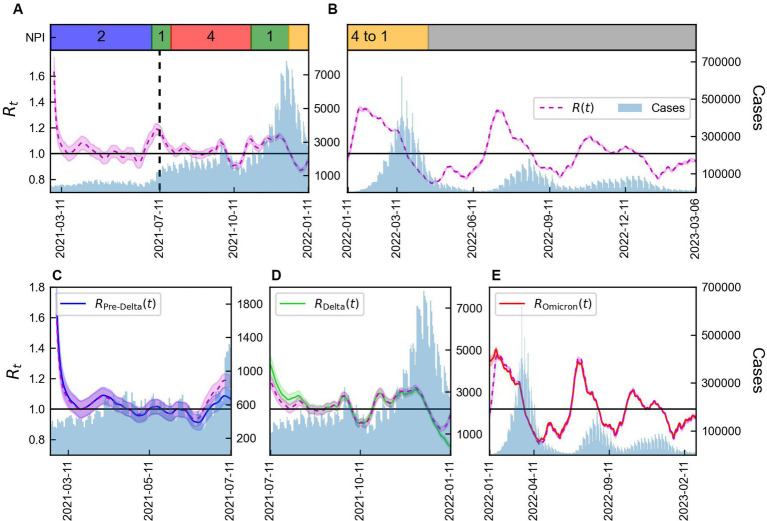
Time-varying reproduction number by variants considering immunity. **(A,B)** Total number of COVID-19 cases (blue) along with the reproduction number using Eq. (2) (magenta line) and NPI levels implemented in Korea. **(C–E)** Total number of COVID-19 cases (blue) along with reproduction number obtained using Eqs. (1) and (2) for Pre-Delta, Delta, and Omicron. The shaded area indicates 95% CrIs and the vertical line represents the start of Delta (T_Delta_).

The values of $R_{t}$ calculated using Eqs. (2) and (3), distinguished as $R\left(t\right)$ and $R_{x}\left(t\right)$ , respectively, were compared, as presented in Figure 3. When $R\left(t\right)$ was calculated based on the total number of cases, significant differences in values for each variant were observed. Calculated $R\left(t\right)$ values using data from February 26, 2021 to January 10, 2022 and from January 11, 2022 to March 6, 2023 are presented in Figures 3A,B, respectively. Supplementary Table S2 summarized the NPI levels implemented in Korea. It is shown in Figures 3A,B, along with the COVID-19 confirmed cases. During the Pre-Delta period with NPI level 2, $R\left(t\right)$ was consistently around 1. Subsequently, a point where $R\left(t\right)$ exceed 1, we could interpret that it is closely related to the emergence of the Delta variant. This relationship is evident from the discrepancies between $R\left(t\right)$ and $R_{x}\left(t\right)$ shown in Figure 3C. Furthermore, during this period, the reduction of the NPI level to 1 led to an increase in $R\left(t\right)$ to 1.2. From July 11, 2021 onwards, $R\left(t\right)$ decreased and remained at approximately 1. It believed that this was a consequence of the NPI level being intensified to 4. After the spread of the Omicron variant in 2022, $R\left(t\right)$ increased to 1.4. On July 11, 2021, $R_{x}\left(t\right)$ for Pre-Delta was lower than $R\left(t\right)$ calculated using the total number of cases, while $R_{x}\left(t\right)$ for Delta was higher. Starting from January 11, 2022, when the Omicron variant accounted for more than 50% of the total cases, $R_{x}\left(t\right)$ for Delta showed a significant decrease compared to $R\left(t\right)$ calculated based on the total number of cases using Eq. (2), as shown in Figure 3D. Although the overall value of $R\left(t\right)$ increased, analysis of $R_{x}\left(t\right)$ , specifically for Delta, revealed a decrease. Thus, despite the overall increase in transmission of the virus in the population, the measures implemented to control the Delta variant were effective in reducing its spread. The NPI intensity was gradually reduced in a phased restoration of daily life, leading to the lifting of social distancing after April 18, 2022. During the Omicron period, $R\left(t\right)$ , exhibited higher volatility than other periods, shown in Figure 3E.

### 3.3 Time-varying reproduction number by variants

**Figure 4:**
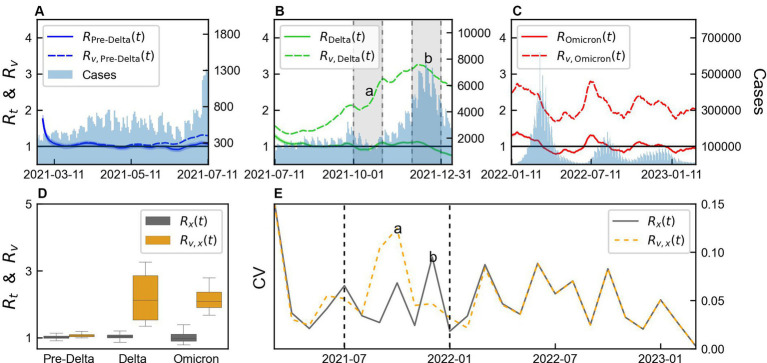
Comparison of variability of time-varying reproduction number by variants considering immunity. **(A–C)** Comparison of time-varying reproduction number with immunity ( $R_{x}\left(t\right)$ ) and without immunity ( $R_{v,x}\left(t\right)$ ), for each variant *x*. **(D)** Box plot of $R_{x}\left(t\right)$ and $R_{v,x}\left(t\right)$ . **(E)** Monthly coefficient of variation (CV) of $R_{x}\left(t\right)$ and $R_{v,x}\left(t\right)$ “a” and “b” represents the points with large differences in CV between $R_{x}\left(t\right)$ and $R_{v,x}\left(t\right)$ . The shaded area in panel **(B)** presents months “a” and ‘b’. The values of “a” and “b” in panel **(E)** indicate estimation from the data within the shaded regions in panel **(B)**. Vertical lines indicate the start Delta and Omicron.

The values of $R_{t}$ and $R_{v}$ calculated for with and without immunity from variant *x* using Eqs. (3) and (5), distinguished as $R_{x}\left(t\right)$ and $R_{v,x}\left(t\right)$ , respectively, are shown in Figure 4. The monthly mean, SD, and CV of $R_{x}\left(t\right)$ and $R_{v,x}\left(t\right)$ are presented in Supplementary Table S3, while the statistics of their estimated values are summarized in Supplementary Table S4. The difference between $R_{x}\left(t\right)$ and $R_{v,x}\left(t\right)$ was not large because the vaccination coverage was not high during Pre-Delta period, as shown in Figure 4A. $R_{x}\left(t\right)$ considering immunity remained approximately 1, as shown in Figure 4B, and after December 31, 2021, it decreased to a value below 1. However, $R_{v,x}\left(t\right)$ without immunity, always remained greater than 1 during Delta. As shown in Figure 4C, $R_{x}\left(t\right)$ was less than 1 for some data points, whereas $R_{v,x}\left(t\right)$ always remained greater than 1, during Omicron. The boxplot of $R_{x}\left(t\right)$ and $R_{v,x}\left(t\right)$ for each variant is shown in Figure 4D. During Pre-Delta, a small difference existed between $R_{x}\left(t\right)$ and $R_{v,x}\left(t\right)$ , while a significant difference was observed during Delta and Omicron. Thus, a large variability existed for each variant, such that a high variability was observed during Delta, which subsequently decreased during Omicron.

CV was calculated from the monthly mean and SD of $R_{x}\left(t\right)$ and $R_{v,x}\left(t\right)$ and presented in Figure 4E. A small difference was observed between the CV calculated during Pre-Delta and Omicron, but a notable difference was observed during Delta. We defined 2 months with significant differences as “*a*” and “*b*,” which were calculated from the shaded area in Figure 4B. During “*a*,” $R_{v,x}\left(t\right)$ exhibited higher variability compared to $R_{x}\left(t\right)$ , accompanied by a substantial increase in $R_{v,x}\left(t\right)$ . Conversely, during “*b,*” $R_{x}\left(t\right)$ showed higher variability compared to $R_{v,x}\left(t\right)$ . Although the magnitudes of the changes were similar, smaller values of $R_{x}\left(t\right)$ resulted in larger variability. This period corresponded to the initiation of a third-dose vaccination campaign. Overall, the variability between $R_{x}\left(t\right)$ and $R_{v,x}\left(t\right)$ differed across the variants, with Delta characterized by significant differences and the start of the third-dose vaccination campaign.

### 3.4 Impact of variants on time-varying reproduction number by regions

**Figure 5:**
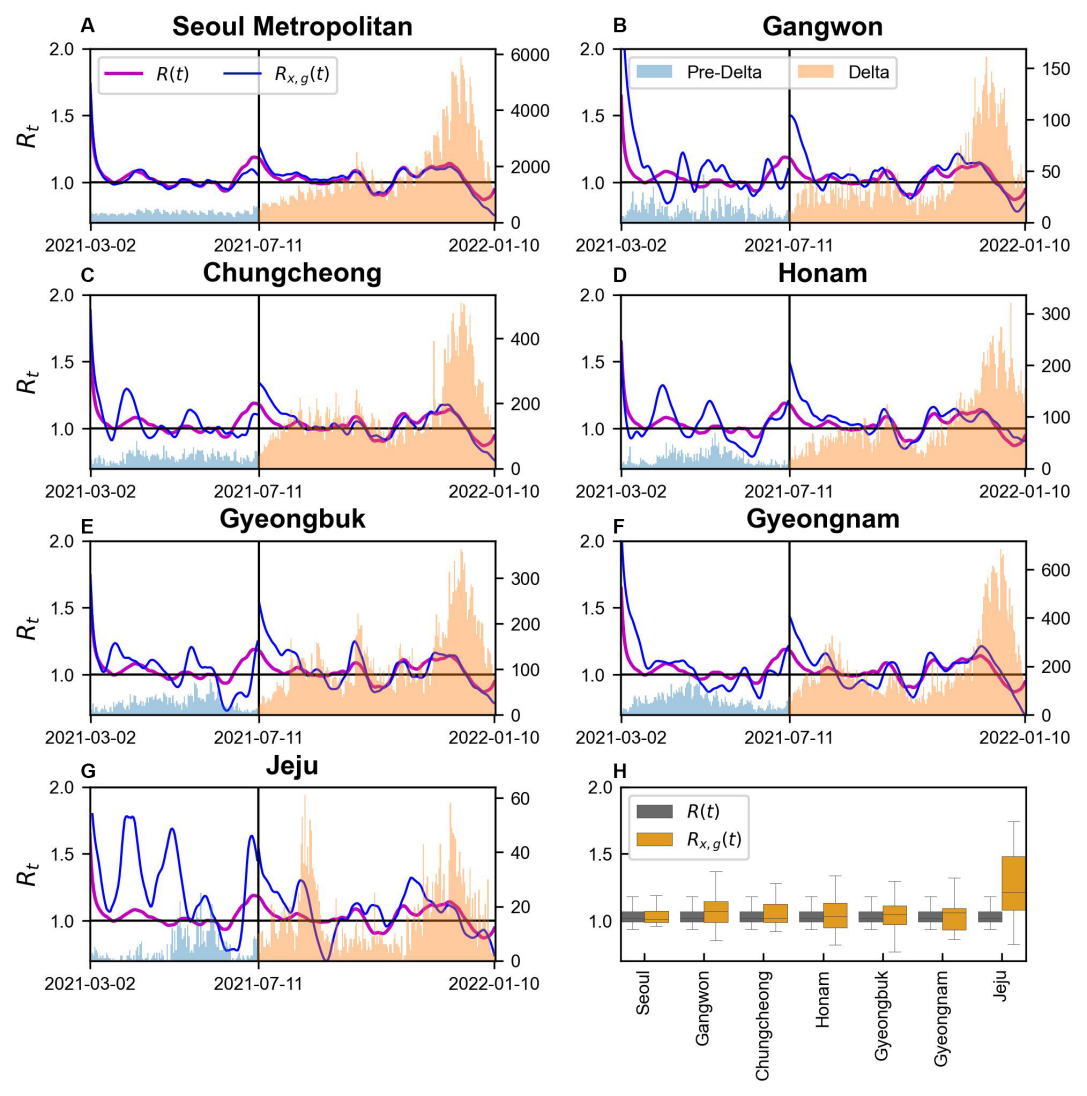
$R\left(t\right)$ and $R_{x,g}\left(t\right)$ in regions during Pre-Delta and Delta **(A–G)** Seoul Metropolitan Area, Gangwon, Chungcheong, Honam, Gyeongbuk, Gyeongnam, and Jeju, respectively.

We conducted a comparison of $R\left(t\right)$ using Eq. (2) and $R_{x,g}\left(t\right)$ using Eq. (6) with the results illustrated in Figure 5. The location of each region in Korea is illustrated in Supplementary Figure S1. Figures 5A–G presents the results for Seoul Metropolitan Area, Gangwon, Chungcheong, Honam, Gyeongbuk, Gyeongnam, and Jeju. Figure 5H shows a regional box plot comparing $R\left(t\right)$ and $R_{x,g}\left(t\right)$ . The discrepancy between $R\left(t\right)$ and $R_{x,g}\left(t\right)$ is the largest in Jeju, while it is almost negligible in Seoul Metropolitan Area. In all regions, a difference between $R\left(t\right)$ and $R_{x,g}\left(t\right)$ is observed around July 11, 2021, coinciding with the transition from the Pre-Delta to the Delta variant. During this period, Seoul Metropolitan Area, Chungcheong, Honam, and Gyeongnam exhibited similar patterns in $R\left(t\right)$ and $R_{x,g}\left(t\right)$ , whereas Gangwon, Gyeongbuk, and Jeju displayed divergent patterns. Particularly in Jeju, $R_{x,g}\left(t\right)$ exhibits significant volatility during the Pre-Delta period, which is likely attributed to the small number of cases, and Gangwon and Gyeongbuk require additional analysis.

**Figure 6:**
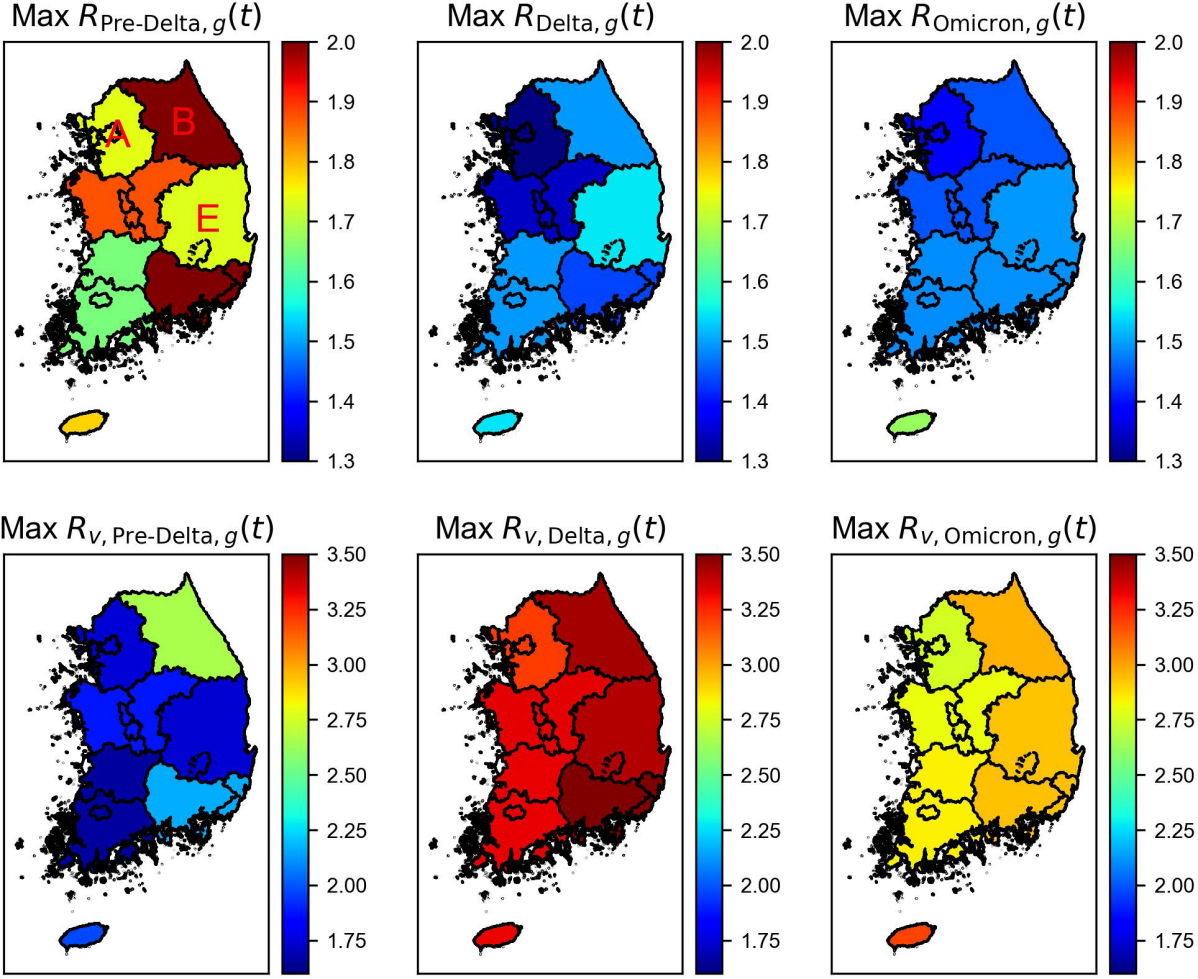
Maximum time-varying reproduction number with the immunity ( $R_{x,g}\left(t\right)$ ) and without immunity ( $R_{v,x,g}\left(t\right)$ ) by regions and variations. Regions A, B, and E indicate Seoul Metropolitan Area, Gangwon, and Gyeongbuk areas of Korea.

We computed $R_{x,g}\left(t\right)$ with immunity using Eq. (6) and $R_{v,x,g}\left(t\right)$ without immunity using Eq. (7) in seven regions of Korea, as aforementioned for Pre-Delta, Delta, and Omicron is shown in Supplementary Figures S4–S6, respectively. A comparison between the calculated maximum value of $R_{x,g}\left(t\right)$ and $R_{v,x,g}\left(t\right)$ by region g is presented in Figure 6. While comparing the $R_{x,g}\left(t\right)$ results for Pre-Delta, Delta, and Omicron, the severity of the variant viruses was difficult to determine. However, while examining the $R_{v,x,g}\left(t\right)$ results, the overall $R_{v,x,g}\left(t\right)$ values were higher during Delta, particularly in Gyeongnam and Gangwon. During Omicron, the $R_{v,x,g}\left(t\right)$ values were higher than those during Pre-Delta. The mean values of $R_{x,g}\left(t\right)$ and $R_{v,x,g}\left(t\right)$ are shown in Supplementary Figure S7.

**Figure 7:**
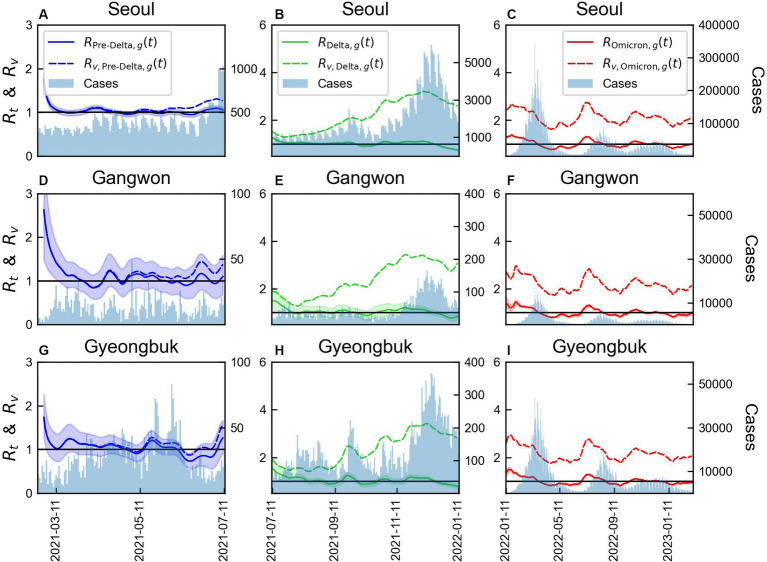
Comparison of time-varying reproduction numbers by variants with or without immunity in three regions. The bars represent the number of COVID-19 cases over time in each region. The line colors correspond to the variants: **(A-C)** Seoul Metropolitan Area, **(D-F)** Gangwon area, **(G-I)** Gyeongbuk area.

The results for three regions, Seoul Metropolitan Area (Region A), Gangwon (Region B), and Gyeongbuk (Region E), were compared, as shown in Figure 7. Variations in the magnitudes of $R_{v,x,g}\left(t\right)$ across different variant periods were observed. Without considering immunity, the mean of $R_{v,x,g}\left(t\right)$ consistently increased over time in all regions. However, a significantly higher value of maximum $R_{v,x,g}\left(t\right)$ compared to an average value of $R_{v,x,g}\left(t\right)$ in each variant period suggested the occurrence of an event that led to a spike in cases in that region. During Pre-Delta, when the increase in the number of cases was relatively smaller compared to that during Delta and Omicron, the difference between the average and maximum values of $R_{v,x,g}\left(t\right)$ was relatively small. Supplementary Table S5 summarizes the maximum, mean, and minimum reproduction numbers for each period by region.

The values of monthly CV of $R_{x,g}\left(t\right)$ and $R_{v,x,g}\left(t\right)$ and vaccination coverage for the three regions are shown in Supplementary Figure S9. Supplementary Table S6 provides a summary of the monthly CV for the two indicators of reproduction number. Supplementary Table S7, on the other hand, outlines Distribution of age population size and vaccination coverage by region. During Pre-Delta, $R_{x,g}\left(t\right)$ and $R_{v,x,g}\left(t\right)$ exhibited similar patterns. However, during the second phase, when the Delta variant spread, a significant variability in $R_{v,x,g}\left(t\right)$ was observed. This period coincided with a rapid increase in the administration of the second and third vaccine doses, indicating an increase in immunity. In the third phase, a consistent pattern in CV across regions was observed, suggesting a similar trend. The number of individuals receiving the third vaccine dose significantly decreased, and owing to an already high number of infected individuals, the difference between $R_{x,g}\left(t\right)$ and $R_{v,x,g}\left(t\right)$ was reduced. In Seoul Metropolitan Area and Gyeongbuk, shown in Supplementary Figure S9, a rapid increase in the number of COVID infected individuals was observed in mid-September 2021. Thus, the variability in CV increased to approximately 0.2.

## 4 Discussion

During outbreak of infectious diseases such as SARS-CoV-2, authorities must accurately monitor the situation to make effective decisions. Factors such as the scale of the epidemic and its spatiotemporal dynamics determine the risk of exposure, pressurize crucial infrastructure, and burden society with diseases. As COVID-19 spread globally, countries adopted various strategies, often following more relaxed measures. Assessing the influence of unique political strategies on disease spread and predicting the outcomes of potential alternative measures are important.

In this study, we investigated the transmission dynamics of COVID-19 by considering its variants and the impact of vaccination coverage on immunity. Our findings aligned with those of previous studies (2, 51, 52), confirming that the Delta variant had the highest number of cases, followed by the Omicron variant. Additionally, the estimated SIs were 3.85, 4.29, 3.56 days for the total period, Pre-Delta, and Delta, respectively, as shown in Figure 2. The estimated SI of the Delta variant further supported the results. Our predictions aligned with the findings of our previous study, as the estimated mean SI of 3.56 days was similar to 3.5 days of our previous finding, 3.7 days of (2) and 3.00 days of (51). This consistency in estimated values of SI further supported the robustness and reliability of the analysis. By understanding the duration between symptom onset in infectors and infectees, we can gain insight into the transmission dynamics of the COVID-19 epidemic and improve public health interventions aimed at controlling the spread of the virus.

The decreasing value of $R_{x}\left(t\right)$ for the Delta variant indicated successful mitigation of virus transmission by interventions, such as increased vaccination coverage and other control measures (Figure 3). This finding underscored the importance of targeted efforts to curb the spread of specific variants, as distinct transmission dynamics exist when compared with the overall epidemic. During Delta, if the vaccination coverage was low (Figure 4), $R_{v,x}\left(t\right)$ would likely have remained above 2. Conversely, the significant decrease in $R_{x}\left(t\right)$ could be attributed to the immunity gained through vaccination. This finding highlighted the substantial benefit of vaccination in reducing the transmission potential of the Delta variant. On comparison of time-varying reproduction numbers by region (Figure 7), the immunity in $R_{x,g}\left(t\right)$ provided a better explanation for the characteristics of the variants and regional differences. If there was no immunity, a significant increase in $R_{v,x,g}\left(t\right)$ could be interpreted.

However, during Pre-Delta period, the gap between the $R_{x}\left(t\right)$ and $R_{v,x}\left(t\right)$ values was minimum, attributed to the low vaccination coverage. Furthermore, though additional vaccinations were administered infrequently during the Omicron period, the impact of immunity persisted due to the high vaccination coverage achieved during the Delta period. These findings underscored the crucial role of vaccination in reducing the spread of COVID-19, and the significance of achieving high vaccination coverage to maximize the benefits of immunity in controlling variant-driven epidemics. Thus, in this study, we emphasize the critical role of vaccination in reducing the risk of infection.

We incorporated the regional heterogeneity and age distribution of the population, as well as the evolving variants and vaccination rates over time, into our calculations of the reproduction number. However, the broader applicability of our results is constrained. The diversity in spatial heterogeneity and human behaviors, which are pivotal to the transmission dynamics of COVID-19, vary across different areas. Therefore, including a variety of populations and environments is crucial to deepen the understanding of the transmission dynamics on a global scale (53–56). Badr et al. (57) identified a statistically significant positive correlation between human mobility patterns and COVID-19 case trends, with a 5–6 days lag reflecting in the reproduction number (R_*t*_). Additionally, several studies have addressed the effect of spatial heterogeneity (55, 56). For instance, Ogwara et al. (55) estimated the time-dependent R_*t*_ for SARS-CoV-2 within Georgia and its health districts using daily case data, akin to our methodology. However, other investigations have estimated $R_{t}$ by accounting for movement and mobility between regions (58). Beyond these methods, the reproductive number can also be determined using various other techniques, such as machine learning algorithms or multi-agent simulations (59, 60).

In addition, the availability of SI data during Omicron was limited due to the rapid and widespread transmission of COVID-19. Therefore, we assumed that the distribution of the SIs during Delta was similar to that during Omicron. Although this assumption introduces some uncertainty, it is essential for the estimation of SI for the entire study period. If the data of infector–infectee pairs were available during Omicron, a more accurate understanding of transmission dynamics during that time could have been provided.

However, despite these limitations, our study is significant as it provides a novel analysis of the impact of variants, immunity, age, and geographical factors on the time-varying reproduction number. In previous studies (27, 55, 61), reproduction numbers were computed using variants or regions. However, the effects of immunity are yet to be considered. To the best of our knowledge, our study is a first to comprehensively examine the influence of such variables on time-varying reproduction numbers at a granular level. Considering the differential effects of variants and immunity across age groups and regions, our study offers valuable insights into the complex dynamics of the COVID-19 pandemic. Overall, this study provides valuable insights into the transmission dynamics of COVID-19 by considering the variants and vaccination.

## 5 Conclusion

Considering the well-established concept of $R_{t}$ (25–27), we have proposed a modified reproduction number that takes into account various data sets: (i) variant-specific $R_{x}\left(t\right)$ , and (ii) $R_{v}\left(t\right)$ , which does not consider immunity, compared with the traditional $R_{t}$ to analyze the effects of immunity. Rather than evaluating those several formulas for $R_{t}$ , our study emphasizes the capability of the proposed reproduction numbers to capture important factors like vaccination and variants, by introducing the several reproductions, namely $R_{v}\left(t\right)\;\mathrm{and}\;R_{x}\left(t\right)$ . Our study highlights the dominance of the Delta variant, effectiveness of vaccination in reducing transmission, and significance of targeted interventions and high vaccination coverage in controlling COVID-19. Despite limitations, our findings improve our understanding of the transmission dynamics of this disease.

## Data availability statement

The original contributions presented in the study are included in the article/Supplementary material, further inquiries can be directed to the corresponding author.

## Ethics statement

Ethical approval was not required for the study involving humans in accordance with the local legislation and institutional requirements because the datasets used in this study are fully anonymized and do not contain any identifiable or personal information.

## Author contributions

GJ: Formal analysis, Methodology, Software, Writing – original draft. JK: Data curation, Formal analysis, Writing – original draft. YL: Data curation, Formal analysis, Writing – original draft. CS: Data curation, Formal analysis, Writing – original draft. KK: Data curation, Formal analysis, Writing – original draft. HL: Conceptualization, Investigation, Methodology, Supervision, Writing – original draft, Writing – review & editing.
