# Supplementary material for: Analysis of the impact of COVID-19 variants and vaccination on the time-varying reproduction number: statistical methods
Source: Front Public Health. 2024 Jul 3;12:1353441. doi: 10.3389/fpubh.2024.1353441 (PMC11253806; doi:10.3389/fpubh.2024.1353441)
Supplement: Supplementary file 1 [file Data_Sheet_1.pdf]

# Supplementary Information

Geunsoo Jang[1], Jihyeon Kim[2], Yeonsoo Lee[2], Changdae Son[2], Kyeong Tae Ko[2], and Hyojung Lee[2,*]

[1]Nonlinear Dynamics and Mathematical Application Center, Kyungpook National University, Daegu, 41566, Republic of Korea
[2]Department of Statistics, Kyungpook National University, Daegu, 41566, Republic of Korea

Corresponding Author:
Hyojung Lee
Department of Statistics, Kyungpook National University, Daegu, 41566, Republic of Korea
Email: hjlee@knu.ac.kr

**Table S1.** COVID-19 reported cases and vaccination rate during the time period by variants.

| Time period | COVID-19 reported cases | Vaccination coverage (%) | | |
|---|---|---|---|---|
| | | $1^{th}$ | $2^{nd}$ | $3^{rd}$ |
| $T_{Pre\text{-}Delta}$ | 74,052 | 30.74 | 9.66 | 0.00 |
| $T_{Delta}$ | 491,010 | 86.24 | 80.93 | 43.90 |
| $T_{Omicron}$ | 29,847,923 | 87.55 | 83.79 | 69.05 |

$T_{Pre\text{-}Delta}$: February 26, 2020 – July 10, 2021

$T_{Delta}$: July 11, 2021 – January 10, 2022

$T_{Omicron}$: January 11, 2022 – March 6, 2023

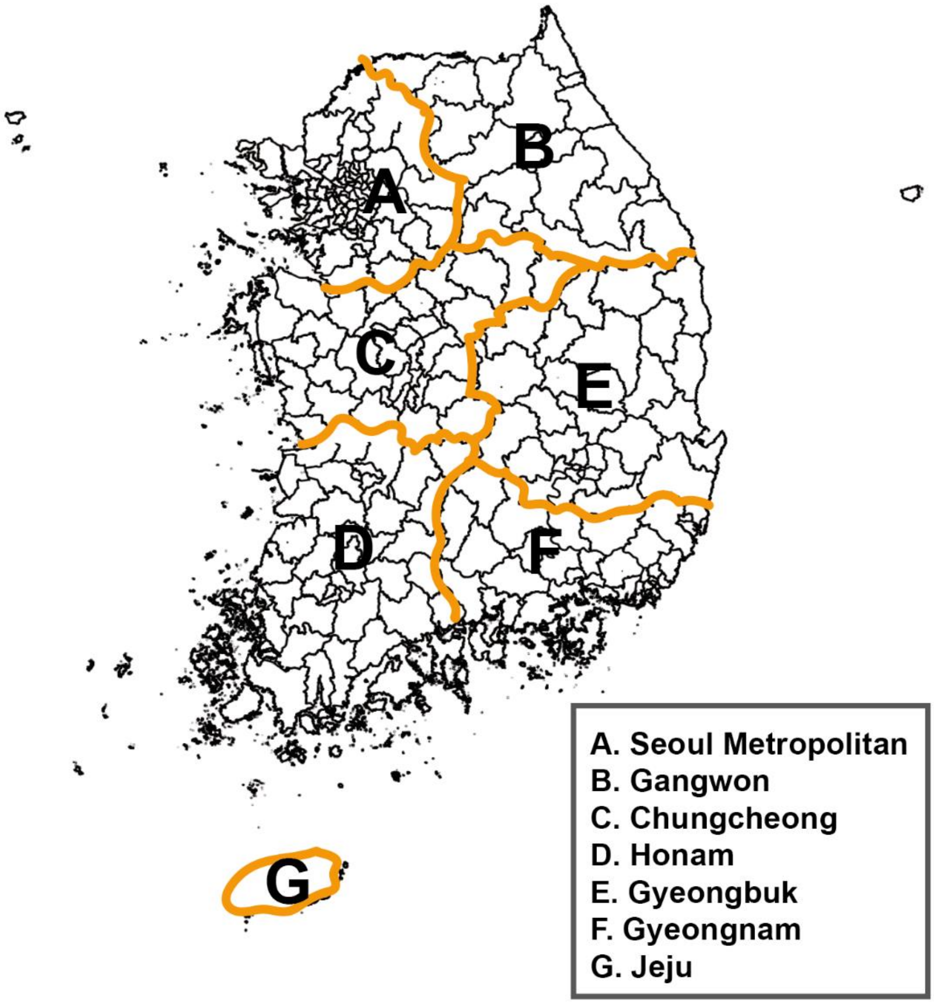


**Fig. S1. Map of Korea illustrating the regional divisions.** The regions are delineated and labeled from A to G. Each region is highlighted by orange lines to emphasize the geographical boundaries.

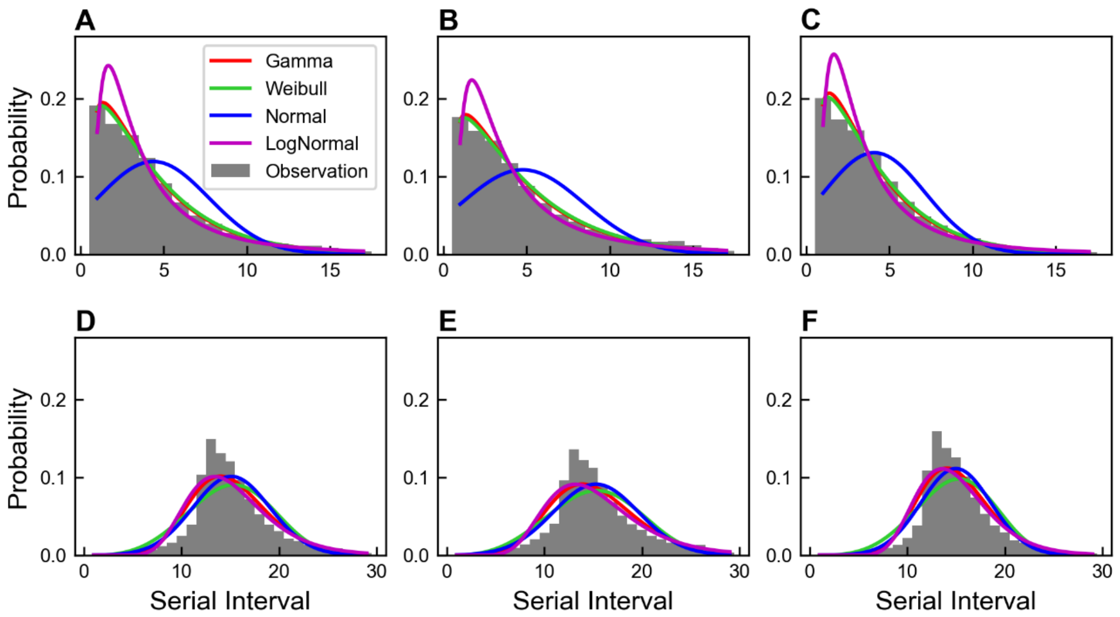


**Fig. S2. Estimated serial interval distribution of COVIS-19 in Korea by time period.** Bars indicate the observed data of serial interval. Truncated distribution during (A) total period, (B) Pre-Delta, and (C) Delta. Shifted distribution during (D) total period, (E) Pre-Delta, and (F) Delta.

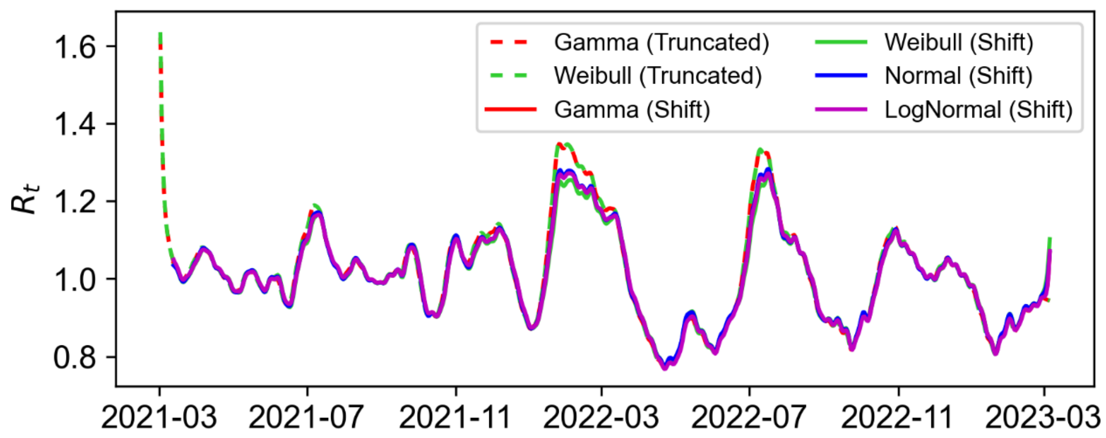


**Fig. S3. Time-varying reproduction number by serial interval distribution.**

**Table S2.** Summary of non-pharmaceutical intervention (NPI) levels implemented in Korea from February 26, 2021, to March 6, 2023 [1].

| Period | NPI level | Description |
|---|---|---|
| 2021-02-26 ~ 2021-06-30 | 2 | Private gatherings allowed up to 8 individuals |
| 2021-07-01 ~ 2021-07-24 | 1 | No restrictions on private gatherings |
| 2021-07-25 ~ 2021-10-31 | 4 | Allow private gatherings up to four people |
| 2021-11-01 ~ 2021-12-18 | 1 | Private gatherings can be held with up to 99 individuals |
| 2021-12-18 ~ 2022-04-18 | 4 to 1 | Gradual restoration of daily life |
| 2022-04-18 ~ 2023-03-06 | - | Lifting of social distancing after April 18, 2022 |

**Table S3.** Values of $R_x(t)$ and $R_{v,x}(t)$ by month

| Month | Variant | $R_x(t)$ in equation (2) | | | $R_{v,x}(t)$ in equation (4) | | |
|---|---|---|---|---|---|---|---|
| | | Mean | SD | CV | Mean | SD | CV |
| 2021-03 | Pre-Delta | 1.110 | 0.171 | 0.154 | 1.116 | 0.170 | 0.152 |
| 2021-04 | Pre-Delta | 1.042 | 0.038 | 0.036 | 1.059 | 0.032 | 0.030 |
| 2021-05 | Pre-Delta | 0.986 | 0.021 | 0.021 | 1.034 | 0.025 | 0.024 |
| 2021-06 | Pre-Delta | 0.972 | 0.040 | 0.042 | 1.121 | 0.061 | 0.055 |
| 2021-07 | Pre-Delta | 1.070 | 0.015 | 0.066 | 1.291 | 0.021 | 0.052 |
| 2021-08 | Delta | 1.141 | 0.075 | 0.034 | 1.411 | 0.074 | 0.036 |
| 2021-09 | Delta | 1.038 | 0.036 | 0.028 | 1.449 | 0.052 | 0.104 |
| 2021-10 | Delta | 1.033 | 0.028 | 0.069 | 1.874 | 0.195 | 0.125 |
| 2021-11 | Delta | 0.974 | 0.067 | 0.024 | 2.282 | 0.286 | 0.045 |
| 2021-12 | Delta | 1.079 | 0.026 | 0.095 | 2.934 | 0.132 | 0.047 |
| 2022-01 | Omicron | 1.018 | 0.097 | 0.018 | 3.097 | 0.145 | 0.032 |
| 2022-02 | Omicron | 0.795 | 0.027 | 0.034 | 2.723 | 0.047 | 0.022 |
| 2022-03 | Omicron | 1.346 | 0.025 | 0.087 | 2.618 | 0.084 | 0.084 |
| 2022-04 | Omicron | 1.258 | 0.043 | 0.047 | 2.573 | 0.056 | 0.045 |
| 2022-05 | Omicron | 1.078 | 0.094 | 0.036 | 2.268 | 0.190 | 0.036 |
| 2022-06 | Omicron | 0.833 | 0.039 | 0.088 | 1.767 | 0.080 | 0.089 |
| 2022-07 | Omicron | 0.867 | 0.031 | 0.057 | 1.846 | 0.066 | 0.057 |
| 2022-08 | Omicron | 0.927 | 0.082 | 0.070 | 1.978 | 0.175 | 0.070 |
| 2022-09 | Omicron | 1.225 | 0.070 | 0.025 | 2.616 | 0.149 | 0.025 |
| 2022-10 | Omicron | 1.022 | 0.072 | 0.083 | 2.185 | 0.153 | 0.083 |
| 2022-11 | Omicron | 0.877 | 0.022 | 0.033 | 1.877 | 0.046 | 0.033 |
| 2022-12 | Omicron | 1.017 | 0.084 | 0.021 | 2.177 | 0.181 | 0.020 |
| 2023-01 | Omicron | 1.043 | 0.034 | 0.051 | 2.236 | 0.073 | 0.051 |
| 2023-02 | Omicron | 1.019 | 0.021 | 0.026 | 2.186 | 0.045 | 0.026 |
| 2023-03 | Omicron | 0.885 | 0.045 | 0.003 | 1.898 | 0.096 | 0.003 |

Mean and SD represent the monthly average and standard deviation of $R_x(t)$ and $R_{v,x}(t)$ respectively. CV is the monthly coefficient of variation, calculated as SD/mean.

**Table S4.** Statistical comparison between values of $R_x(t)$ and $R_{v,x}(t)$

| | $R_x(t)$ | | | $R_{v,x}(t)$ | | |
|---|---|---|---|---|---|---|
| | Pre-Delta | Delta | Omicron | Pre-Delta | Delta | Omicron |
| Maximum | 1.753 | 1.293 | 1.391 | 1.756 | 3.260 | 2.795 |
| Mean | 1.031 | 1.027 | 1.014 | 1.099 | 2.246 | 2.144 |
| Median | 1.012 | 1.042 | 0.981 | 1.063 | 2.124 | 2.097 |
| Minimum | 0.912 | 0.755 | 0.788 | 0.992 | 1.347 | 1.674 |
| SD | 0.100 | 0.095 | 0.154 | 0.110 | 0.668 | 0.292 |
| CV | 0.097 | 0.092 | 0.152 | 0.100 | 0.298 | 0.136 |
| Proportion of $R > 1$ | 0.614 | 0.703 | 0.476 | 0.947 | 1 | 1 |

CV is the coefficient of variation. SD: standard deviation. The proportion of R>1 indicates the number of time points that satisfy when either $R_x(t)$ or $R_{v,x}(t)$ is greater than 1.

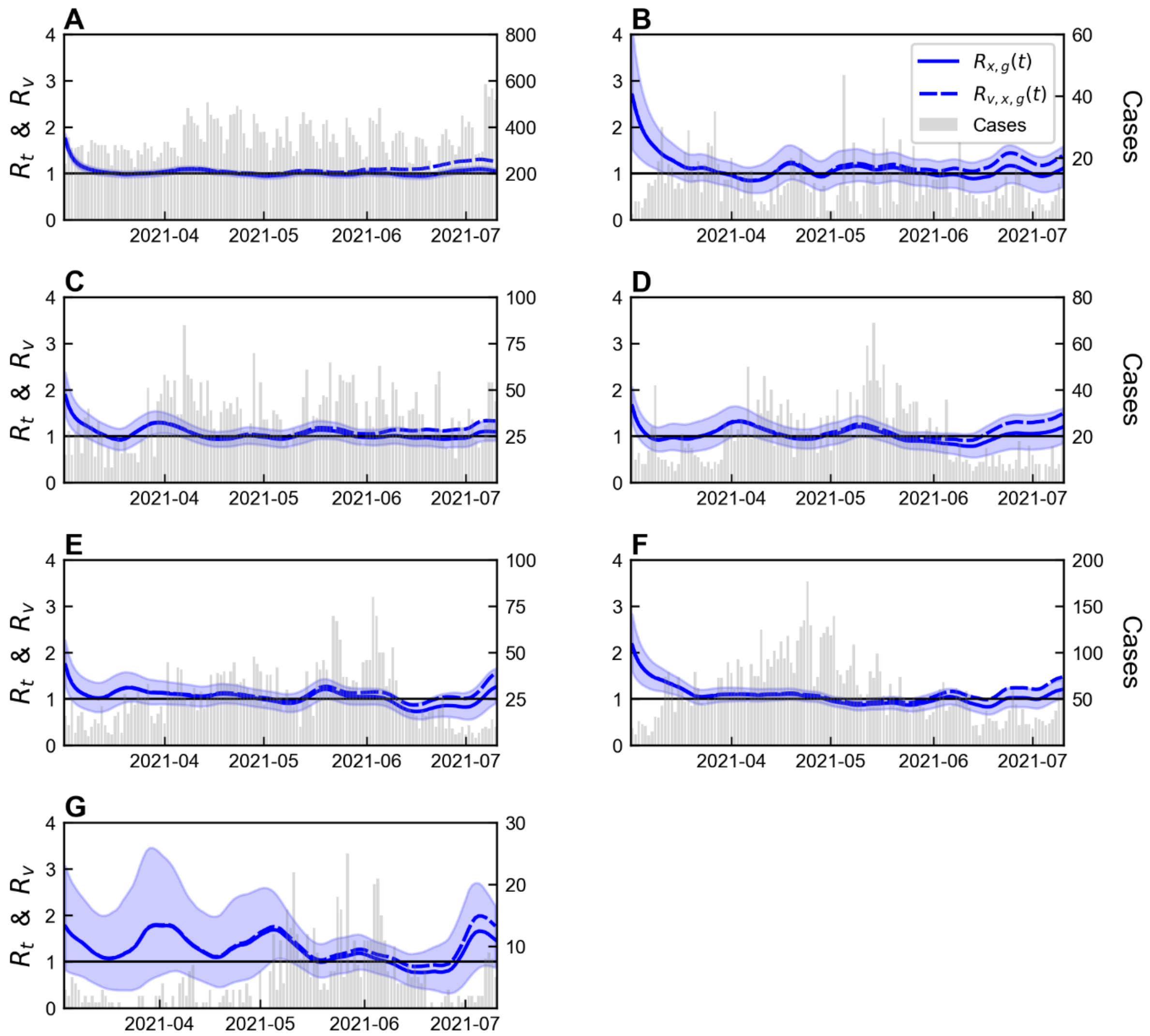


**Fig. S4. Time-varying reproduction number in each region during Pre-Delta.** (**A–G**) Seoul Metropolitan Area, Gangwon, Chungcheong, Honam**,** Gyeongbuk**,** Gyeongnam**,** and Jeju, respectively.

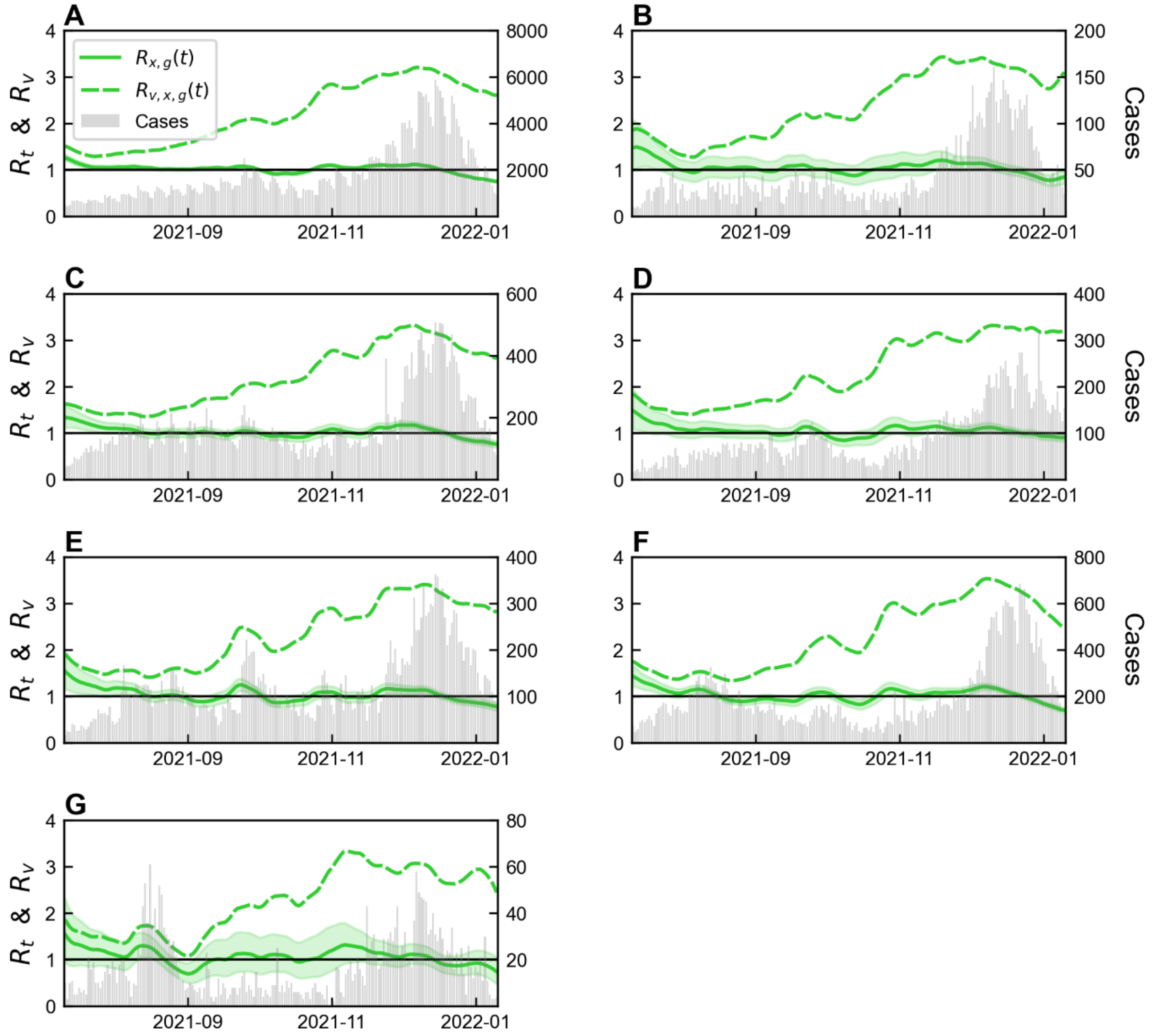


**Fig. S5. Time-varying reproduction number in each region during Delta. (A–G)** Seoul Metropolitan Area, Gangwon, Chungcheong**,** Honam**,** Gyeongbuk**,** Gyeongnam**,** and Jeju, respectively.

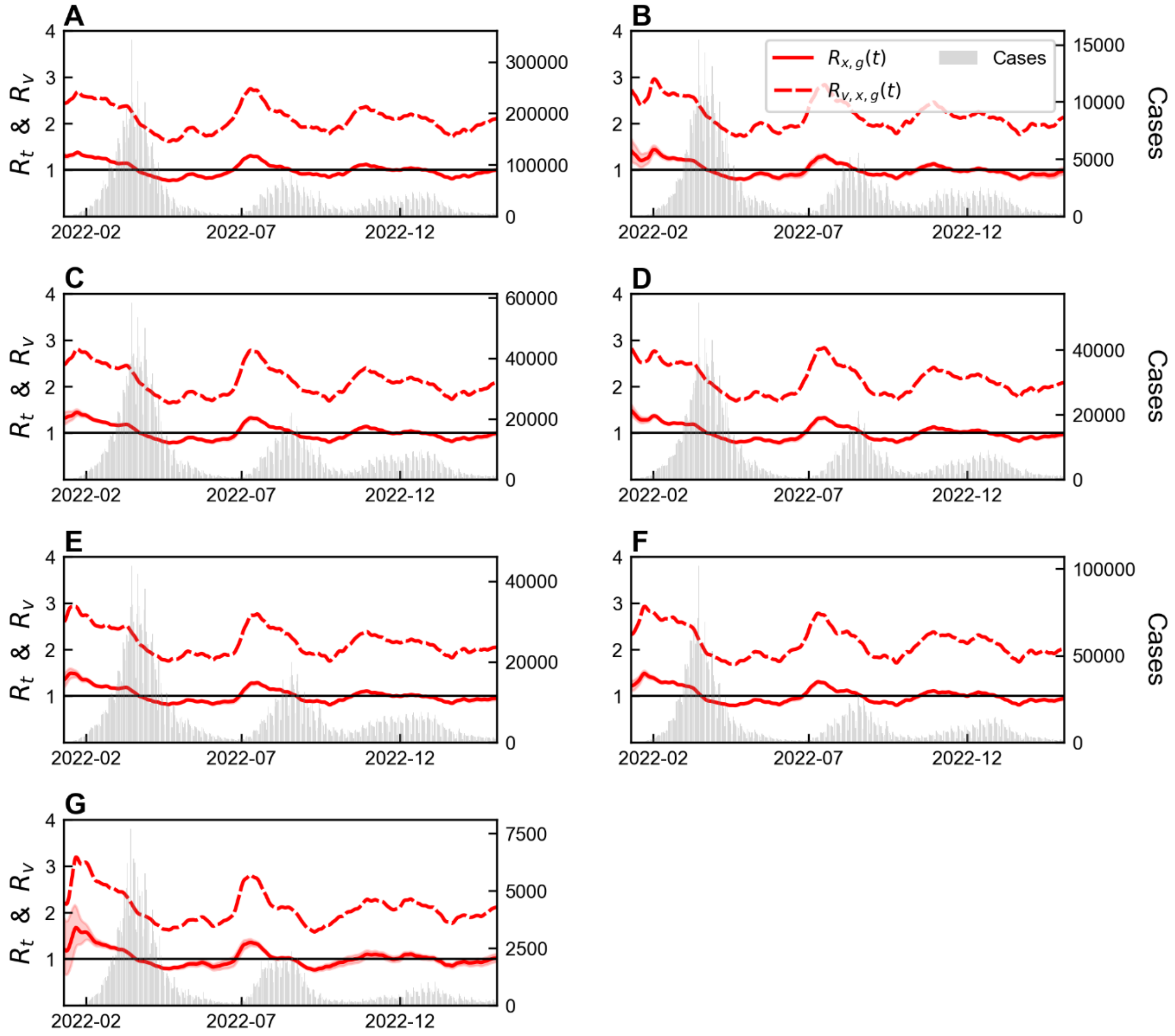


**Fig. S6. Time-varying reproduction number in each region during Omicron.** (**A–G**) Seoul Metropolitan Area, Gangwon, Chungcheong, Honam, Gyeongbuk, Gyeongnam, and Jeju, respectively.

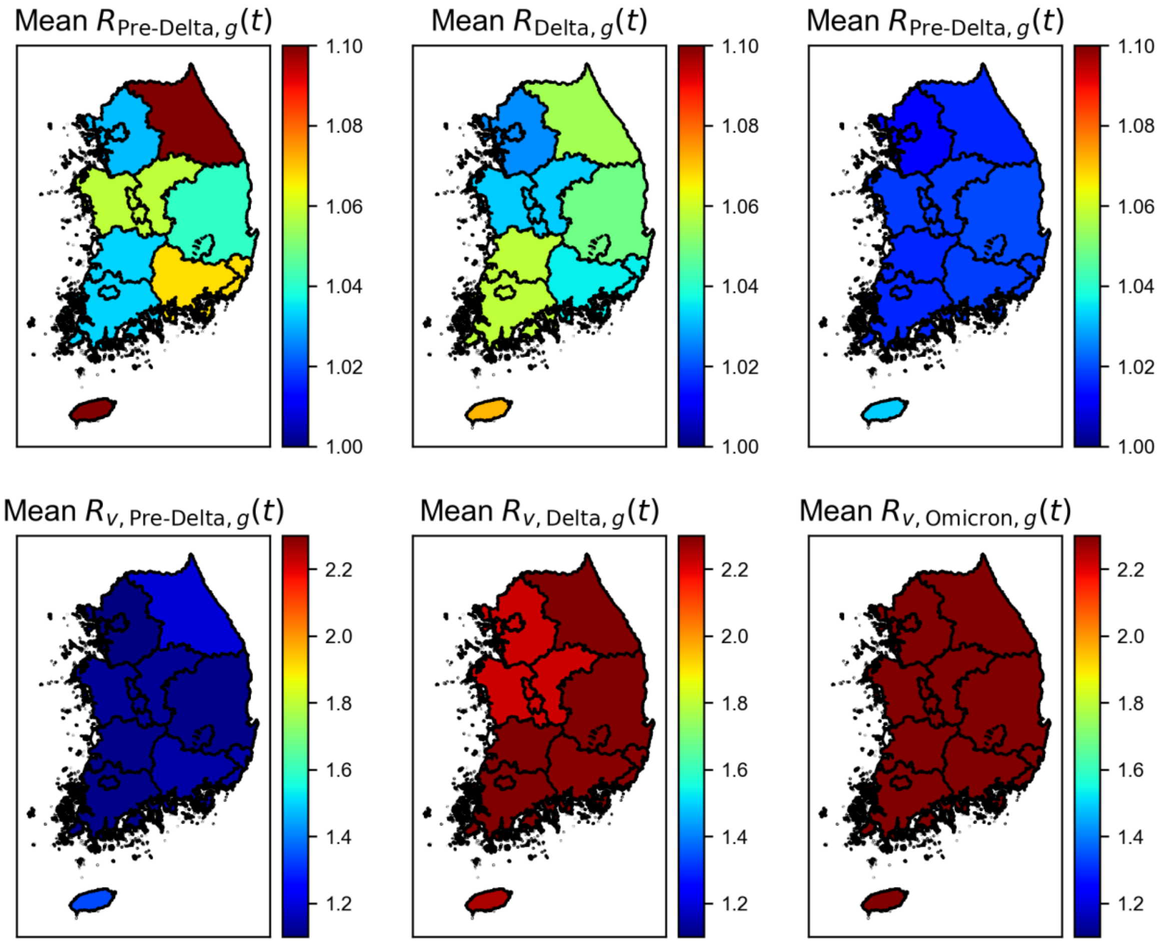


**Fig. S7. Mean of the time-varying reproduction number with immunity ($R_{x,g}(t)$) and without immunity ($R_{v,x,g}(t)$) by regions and variations.**

**Table S5.** $R_{x,g}(t)$ and $R_{v,x,g}(t)$ by region

| Region | Variant | $R_{x,g}(t)$ | | | | $R_{v,x,g}(t)$ | | | |
|---|---|---|---|---|---|---|---|---|---|
| | | Max | mean | min | SD | max | mean | min | SD |
| A | Pre-Delta | 1.739 | 1.031 | 0.948 | 0.096 | 1.742 | 1.095 | 0.983 | 0.112 |
| | Delta | 1.264 | 1.027 | 0.754 | 0.089 | 3.208 | 2.217 | 1.295 | 0.664 |
| | Omicron | 1.383 | 1.012 | 0.772 | 0.152 | 2.752 | 2.109 | 1.616 | 0.287 |
| B | Pre-Delta | 2.569 | 1.105 | 0.841 | 0.255 | 2.573 | 1.188 | 0.850 | 0.248 |
| | Delta | 1.485 | 1.055 | 0.779 | 0.135 | 3.440 | 2.385 | 1.271 | 0.712 |
| | Omicron | 1.445 | 1.016 | 0.803 | 0.155 | 2.965 | 2.182 | 1.733 | 0.302 |
| E | Pre-Delta | 1.737 | 1.041 | 0.736 | 0.152 | 1.740 | 1.111 | 0.871 | 0.131 |
| | Delta | 1.539 | 1.048 | 0.787 | 0.138 | 3.417 | 2.327 | 1.412 | 0.680 |
| | Omicron | 1.493 | 1.020 | 0.817 | 0.158 | 2.930 | 2.170 | 1.756 | 0.299 |

Max, min, and SD indicate the maximum, minimum, and standard deviation during each period in Regions A, B, and E, respectively.

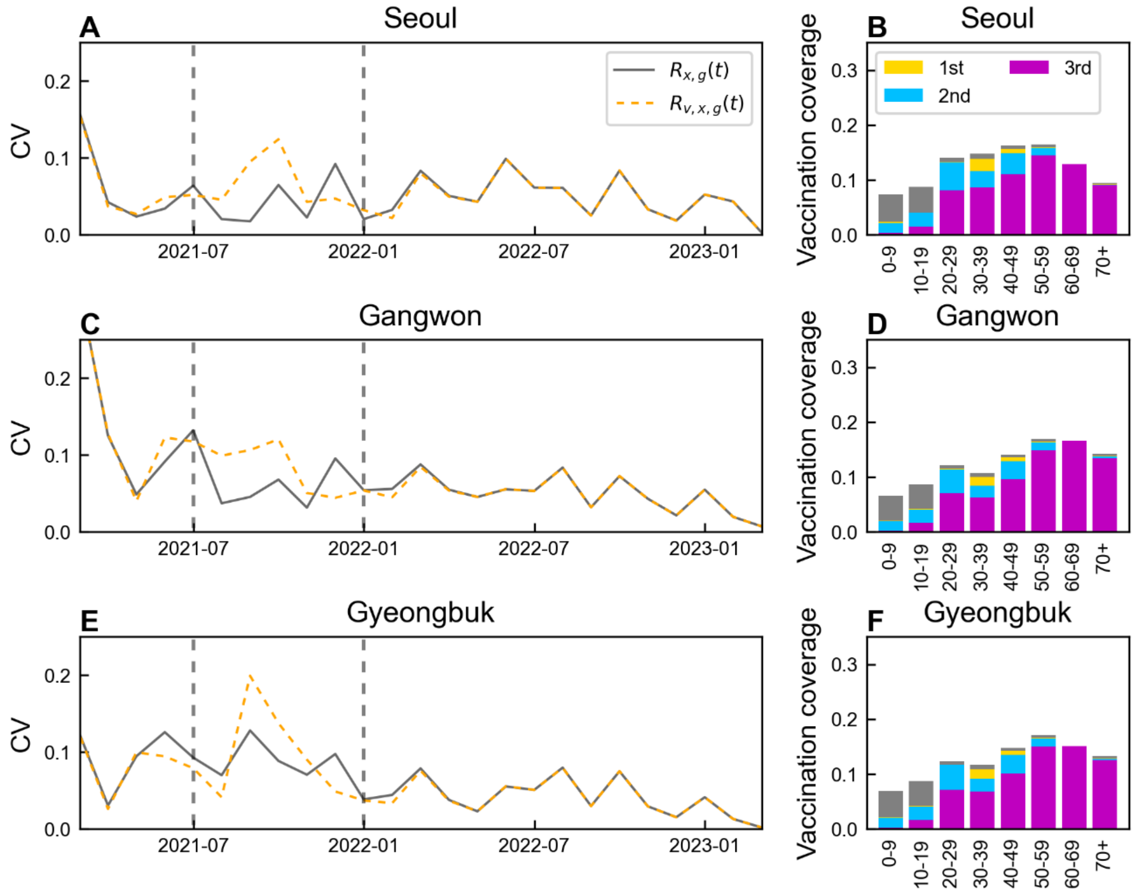


**Fig. S9. Monthly coefficient of variation of $R_{x,g}(t)$ and $R_{v,x,g}(t)$ and vaccination coverage by three regions.** The regions correspond to Seoul metropolitan area, Gangwon, and Gyeongbuk in the upper, middle, and lower panels, respectively. (A, C, E) Monthly coefficient of variation of $R_{x,g}(t)$ (gray) and $R_{v,x,g}(t)$ (orange). (B, D, F). Vaccination coverage of COVID-19 by age groups by region. The dashed vertical lines represent the start of variant spread (Delta, Omicron).

**Table S6.** CV by region

| Month | Region | | | | | |
|---|---|---|---|---|---|---|
| | A | | B | | E | |
| | $R_{x,g}(t)$ | $R_{v,x,g}(t)$ | $R_{x,g}(t)$ | $R_{v,x,g}(t)$ | $R_{x,g}(t)$ | $R_{v,x,g}(t)$ |
| 2021-03 | 0.159 | 0.157 | 0.297 | 0.294 | 0.224 | 0.222 |
| 2021-04 | 0.042 | 0.037 | 0.124 | 0.127 | 0.028 | 0.022 |
| 2021-05 | 0.024 | 0.027 | 0.051 | 0.043 | 0.029 | 0.031 |
| 2021-06 | 0.034 | 0.049 | 0.087 | 0.118 | 0.079 | 0.080 |
| 2021-07 | 0.064 | 0.051 | 0.131 | 0.116 | 0.098 | 0.084 |
| 2021-08 | 0.020 | 0.046 | 0.039 | 0.101 | 0.099 | 0.044 |
| 2021-09 | 0.018 | 0.095 | 0.044 | 0.106 | 0.069 | 0.139 |
| 2021-10 | 0.065 | 0.124 | 0.071 | 0.123 | 0.118 | 0.161 |
| 2021-11 | 0.022 | 0.042 | 0.032 | 0.051 | 0.027 | 0.044 |
| 2021-12 | 0.092 | 0.047 | 0.095 | 0.044 | 0.093 | 0.048 |
| 2022-01 | 0.020 | 0.032 | 0.055 | 0.054 | 0.067 | 0.081 |
| 2022-02 | 0.033 | 0.022 | 0.056 | 0.045 | 0.031 | 0.021 |
| 2022-03 | 0.083 | 0.080 | 0.088 | 0.085 | 0.125 | 0.122 |
| 2022-04 | 0.051 | 0.050 | 0.055 | 0.053 | 0.027 | 0.026 |
| 2022-05 | 0.043 | 0.043 | 0.045 | 0.045 | 0.033 | 0.033 |
| 2022-06 | 0.099 | 0.099 | 0.055 | 0.055 | 0.080 | 0.081 |
| 2022-07 | 0.062 | 0.061 | 0.054 | 0.054 | 0.060 | 0.059 |
| 2022-08 | 0.061 | 0.061 | 0.083 | 0.083 | 0.081 | 0.080 |
| 2022-09 | 0.025 | 0.025 | 0.032 | 0.032 | 0.025 | 0.025 |
| 2022-10 | 0.084 | 0.084 | 0.072 | 0.072 | 0.076 | 0.076 |
| 2022-11 | 0.033 | 0.033 | 0.043 | 0.043 | 0.027 | 0.027 |
| 2022-12 | 0.019 | 0.019 | 0.022 | 0.021 | 0.027 | 0.027 |
| 2023-01 | 0.053 | 0.053 | 0.055 | 0.055 | 0.056 | 0.056 |
| 2023-02 | 0.043 | 0.043 | 0.021 | 0.021 | 0.013 | 0.013 |
| 2023-03 | 0.003 | 0.003 | 0.007 | 0.007 | 0.004 | 0.004 |

**Table S7.** Distribution of age population size and vaccination coverage by region

| **Region** | | **0-9** | **10-19** | **20-29** | **30-39** | **40-49** | **50-59** | **60-69** | **70+** |
|---|---|---|---|---|---|---|---|---|---|
| **A** | 1st vaccine | 0.023 | 0.041 | 0.133 | 0.138 | 0.156 | 0.160 | 0.127 | 0.092 |
| | 2nd vaccine | 0.022 | 0.040 | 0.132 | 0.116 | 0.149 | 0.158 | 0.126 | 0.092 |
| | 3rd vaccine | 0.003 | 0.016 | 0.081 | 0.087 | 0.111 | 0.144 | 0.128 | 0.091 |
| | Proportion of age population | 0.073 | 0.087 | 0.141 | 0.148 | 0.162 | 0.165 | 0.128 | 0.095 |
| **B** | 1st vaccine | 0.021 | 0.041 | 0.115 | 0.101 | 0.136 | 0.165 | 0.164 | 0.138 |
| | 2nd vaccine | 0.020 | 0.041 | 0.114 | 0.085 | 0.129 | 0.163 | 0.163 | 0.137 |
| | 3rd vaccine | 0.003 | 0.016 | 0.070 | 0.063 | 0.096 | 0.149 | 0.166 | 0.135 |
| | Proportion of age population | 0.066 | 0.087 | 0.121 | 0.108 | 0.141 | 0.169 | 0.166 | 0.142 |
| **E** | 1st vaccine | 0.022 | 0.042 | 0.117 | 0.109 | 0.143 | 0.166 | 0.150 | 0.129 |
| | 2nd vaccine | 0.021 | 0.041 | 0.117 | 0.091 | 0.136 | 0.165 | 0.149 | 0.128 |
| | 3rd vaccine | 0.003 | 0.016 | 0.072 | 0.068 | 0.101 | 0.151 | 0.151 | 0.126 |
| | Proportion of age population | 0.069 | 0.087 | 0.124 | 0.117 | 0.148 | 0.171 | 0.151 | 0.133 |

# References

[1] KDCA (The Korea Disease Control and Prevention Agency) Data. https://www.data.go.kr/data/15106451/fileData.do [accessed 2 March 2024]
